# Immunization with recombinant *Streptococcus pneumoniae* PgdA protects mice against lung invasion

**DOI:** 10.3389/ebm.2024.10119

**Published:** 2024-10-14

**Authors:** Jiangming Xiao, Bichen Liu, Yibing Yin, Xuemei Zhang

**Affiliations:** ^1^ Department of Laboratory Medicine, Chongqing General Hospital, Chongqing University, Chongqing, China; ^2^ Department of Laboratory Medicine, Key Laboratory of Diagnostic Medicine (Ministry of Education), Chongqing Medical University, Chongqing, China

**Keywords:** *Streptococcus* pneumoniae, PgdA, vaccine candidate, novel strategy, colonization

## Abstract

Current pneumococcal vaccines, including the pneumococcal polysaccharide (PPV23) and conjugate (PCV13) vaccines, offer protection against specific serotypes but pose risks of serotype replacement that can alter the composition of the nasopharyngeal microbiota. To address this challenge, a novel strategy has been proposed to provide effective protection without disrupting the colonization of other bacterial populations. In our study, we found that subcutaneous immunization with recombinant peptidoglycan N-acetylglucosamine deacetylase A (rPgdA) elicited robust humoral and cellular immune responses, significantly reducing the invasion of *Streptococcus* pneumoniae in the lungs without affecting nasopharyngeal carriage. Furthermore, rPgdA antisera were shown to diminish bacterial invasion of lung epithelial cells *in vitro*. Notably, sera from patients with invasive pneumococcal infections exhibited higher levels of antibodies against the PgdA protein compared to sera from healthy adults, suggesting that a natural immune response to this protein occurs during infection. These results suggest a promising new target for the development of pneumococcal vaccines.

## Impact statement

Long-term vaccination strategies against *Streptococcus pneumoniae* have focused on eliminating asymptomatic carriage and disease, leading to changes in the colonizing microbiota with unknown future consequences. As a result, a novel strategy for providing effective protection while allowing potential bacterial populations, such as *S. pneumoniae*, to colonize has been proposed. In this context, we show that PgdA immunization can significantly reduce pneumococcal invasion of the lungs without affecting carriage. These findings may provide a new candidate protein vaccine for the development of new immunization strategies for pneumococcal diseases.

## Introduction

*Streptococcus pneumoniae* is a natural component of human nasopharyngeal microbiota, but it is also an important pathogen of various respiratory tract illnesses and invasive diseases, including community-acquired pneumonia, sepsis, meningitis, sinusitis and otitis media [1, 2]. Pneumococcal illness affects over one million children under the age of five annually around the world, and the mortality rate is roughly 200,000 [2]. Additionally, *S. pneumoniae* is one of the most significant drug-resistant bacteria in the world and is in urgent need of novel treatments, according to the World Health Organization. Due to the potential life threat of *S. pneumoniae* and the increase of its drug resistance, vaccination has become the most effective method to prevent pneumococcal diseases.

Current vaccines based on the capsular polysaccharide of *S. pneumoniae*, including the 23-valent pneumococcal polysaccharide vaccine (PPV23) and pneumococcal conjugate vaccines (PCVs), have successfully reduced the incidence of invasive pneumococcal diseases and the transmission of vaccine serotypes, providing substantial benefits to public health. However, numerous epidemiological studies have shown that while the introduction of PCVs significantly decreased the nasopharyngeal carriage rate of vaccine-covered serotypes, it also led to a significant increase in the carriage rate of non-vaccine serotypes [3–7]. Additionally, the prevalence of other bacteria, such as *Haemophilus influenzae* and *Staphylococcus aureus*, tends to rise with the introduction of PCVs [8–11]. Research has also demonstrated that the nasopharyngeal microbiota, including *S. pneumoniae*, can prevent the spread of potentially more dangerous pathogens like *Streptococcus pyogenes* and *S. aureus* through competitive rejection [12, 13]. These studies suggest that an indirect effect of PCV vaccination might be the disruption of the nasopharyngeal microbiota, leading to various clinical issues that cannot be disregarded. Consequently, some researchers have proposed a novel immunization strategy to combat pneumococcal disease: a vaccine candidate that can elicit an immune response and prevent the occurrence and development of pneumococcal disease while having no impact on the colonization of potential bacteria in the nasopharynx [14, 15]. Greene et al. verified the potential feasibility of this strategy using pneumococcal surface protein A (PspA) in a pneumococcal disease model caused by influenza virus [14]. Therefore, we aim to explore whether other pneumococcal proteins might have similar protective functions.

Peptidoglycan N-acetylglucosamine deacetylase (PgdA), encoded by the *pgdA* gene, can deacetylate peptidoglycan and make pneumococcus more resistant to host lysozyme, thus increasing the virulence of *S. pneumoniae* [16, 17]. The importance of PgdA for virulence has also been demonstrated in other pathogens, such as *Listeria*, *Enterococcus faecalis*, *Streptococcus suis*, and *Streptococcus iniae* [16, 18–21]. The *pgdA* variants have also been shown to be involved in penicillin resistance [22]. Therefore, some researchers have taken PgdA as an antibacterial target to search for inhibitors that inhibit the activity of PgdA [23, 24]. A recent study showed that PgdA immunization could not reduce the colonization of *S. pneumoniae* TIGR4 strain in the nasopharynx due to capsule factors. However, whether PgdA immunization can protect against invasive pneumococcal disease remains unclear. In addition, our previous study showed that *pgdA* is an *in vivo* inducible gene, and its expression is markedly elevated during lung tissue infection [25], suggesting that it is crucial to the pathogenesis of *S. pneumoniae*. Therefore, in this study, we subcutaneously inoculated recombinant PgdA into mice to study whether the vaccine can effectively prevent the invasive disease of *S. pneumoniae* without impacting the colonization of *S. pneumoniae* in the nasopharynx.

## Materials and methods

### Construction of rPgdA expression plasmid

Since full-length membrane proteins are difficult to obtain, we chose the extracellular domains for expression. The transmembrane region of PgdA was predicted by TMHMM^1^, and amino acids of the extracellular domain at positions 39 to 463 were selected as the sequence of the recombinant protein. The corresponding gene fragment of *pgdA* was amplified by PCR from chromosomal DNA isolated from *S. pneumoniae* TIGR4 with the primers TAG​CTC​TTC​AAA​GCT​TTG​AAG​ATC​TAC​CAG​CAA​AAA​AG and TAC​TCG​AGT​TAT​TCA​TCA​CGA​CTA​TAG​TAC​A using the PrimeSTAR PCR reagent kit (Takara). The following conditions were used for amplification: 94°C for 10 min, 34 cycles of 94°C for 30 s, 58°C for 30 s, 72°C for 10 min, and one final extension step of 72°C for 10 min. Then the PCR fragments were digested with NdeI/XhoI enzymes and cloned into the corresponding restriction sites in pPAL7 with T4 DNA Ligase (New England BioLabs) to generate plasmid pPAL7-PgdA, forming a sequence that encodes a fusion protein of profinity eXact tag-PgdA. The expression vector was finally identified by sequencing.

### Protein expression and purification

The recombinant PgdA (rPgdA) was obtained and purified according to the instruction manual of Profinity eXact™ Protein Purification System (BIORAD). Briefly, the expression plasmid was transformed into BL21 (DE3) for protein expression. The monoclonal colony was selected and cultured in 5 mL of LB supplemented with 100 μg/mL ampicillin until OD_600_ = 0.5 at 37°C with shaking at 180 rpm. Then the activated bacterial solution was added into 500 mL of LB containing 100 μg/mL ampicillin and the mixture was further cultured at 37°C with shaking at 180 rpm until OD_600_ = 0.5. The target protein was induced by shaking at 20°C for about 10 h at a concentration of 0.5 mM isopropyl-β-D-thiogalactopyranoside (IPTG). The cells were harvested by centrifugation followed by wash twice and resuspended with 25 mL of Wash Buffer (0.1 M sodium phosphate, pH 7.2). The bacteria were then destroyed by 25% strength ultrasonic (10 s ON and 10 s OFF) in the ice bath. The supernatant containing the protein was collected by centrifugation followed by sterile filtration with 0.22 µm Millex-GP Filter Unit (Millipore). The chilled (4°C) cell lysate was then loaded into the column packed with 4 mL of Profinity eXact resin and washed with Wash Buffer for three times. After that, the column was incubated at 4°C for overnight with 5 mL of Elution Buffer (0.1 M sodium phosphate, 0.1 M sodium fluoride, pH 7.2) before the tag-free protein was eluted and collected. Endotoxin was removed by the ToxinEraser™ Endotoxin Removal Kit (GenScript). SDS-PAGE and Coomass Bright Blue staining were used to identify protein size and purity.

### Animals

Female 6–8-week-old C57BL/6J mice used in this study were purchased from and raised at Chongqing Medical University, Chongqing, China. All animal experiments involved in this paper were approved by the Animal Care and Use Committee of the Chongqing Medical University.

### Bacterial strains and growth conditions

*S. pneumoniae* type 2 strain D39 was purchased from the National Collection of Type Cultures (London, United Kingdom). *S. pneumoniae* type 4 strain TIGR4 was obtained from the American Type Culture Collection (ATCC; Manassas, VA, United States). The other *S. pneumoniae* strains including serotype 1 (CMCC 31109), serotype 3 (CMCC 31203), serotype 6B (CMCC 31207), serotype 7F (CMCC 31507), serotype 9V (CMCC 31216), serotype 14 (CMCC 31614), serotype 18C (CMCC 31687), serotype 19F (CMCC 31693), serotype 23F (CMCC 31759), were obtained from the China Medical Culture Collection (CMCC; Beijing, China) center. All *S. pneumoniae* strains were grown on Columbia sheep blood agar plates (Chongqing Pangtong, China) or semisynthetic casein hydrolysate medium supplemented with 0.5% yeast extract (C + Y medium) at 37°C with 5% CO_2_. The CFU of pneumococci were calculated by plating on blood agar. The 50% lethal doses (LD50s) of D39 in C57BL/6J mice have been determined to be approximately 50 CFU.

### Immunization and sera collection

Mice were subcutaneously injected with either 35 μg of rPgdA mixed with an equal volume of Inject Alum Adjuvant (Thermo Scientific) or with adjuvant only for three times with an interval of 14 days. Sera were collected before each vaccination and 1 week after the final vaccination and stored at −80°C until further use.

### ELISA

The 96-well plates were coated with 5 μg/mL of rPgdA protein at 4°C for overnight. The plates were then blocked with 2% bovine serum albumin at 37°C for 2 h after being washed 3 times with PBS/T (containing 0.05% Tween-20). After three PBS/T rinses, gradient-diluted antiserum was added to the wells, and the plates were then incubated at 37°C for 1 hour. The HRP-labeled secondary antibody was added at a ratio of 1:5000 after five PBS/T washes. After 45 min of continuing incubation, the plates were washed with PBS/T for 6 times and TMB substrate was added to develop color for 15 min. The absorbance of 450 nm was read after stop solution was added, with 2.1 times of blank control as the threshold. HRP-labeled goat anti-mouse IgG and goat anti-human IgG were purchased from KPL (Gaithersburg, MD, United States). HRP-labeled goat anti-mouse IgG1, IgG2a, IgG2b, and IgG3 subtypes were purchased from Santa Cruz (Santa Cruz Biotechnology, Santa Cruz, CA).

### Western blot analysis

A total of 11 different serotypes of *S. pneumoniae* were collected and lysed by 0.5% deoxycholic scid sodium salt. Whole-cell lysates were separated with 10% SDS-PAGE and transferred to *Immobilon* membranes (Immobilon P, Millipore) electrophoretically. Membranes were blocked for 2 h at room temperature in TBS with 0.1% tween 20 (TBS-T) and 5% skim milk powder. The membranes were washed three times in TBS-T at room temperature and then incubated for overnight in antisera (1: 2000 dilution) at 4°C. Then the membranes were washed with TBS-T and incubated with HRP-conjugated goat anti-mouse secondary antibody diluted to 1:5000 in TBS-T containing 5% skim milk powder. After the membranes were washed with TBS-T, the membranes were developed with HRP substrate luminol reagent (Millipore, MA).

### Bacterial adhesion and invasion of A549 epithelial cells

A549 cells were seeded at 2 × 10^5^ cells per well in a 24 well plate overnight. Pooled sera from the control or rPgdA groups were mixed with TIGR4 bacteria and incubated on a rotator at room temperature for 1 h. After removing the medium from the plate, bacteria (2 × 10^7^) were added to each well and incubated at 37°C with 5% CO_2_. For the adhesion experiment, after 30 min of incubation, the wells were then washed with PBS to remove unattached bacteria. For the invasion experiment, after 2 h of incubation, the wells were then washed with PBS before being treated with penicillin (10 μg/mL) and gentamicin (200 μg/mL) for 15 min to kill extracellular bacteria, after which the cells were washed with PBS again and lysed by adding 100 μL of distilled water to the wells. After serial dilution and plating onto blood agar plates, samples were examined for viable counts after overnight incubation.

### Cytokine assays

Two weeks after the final immunization, the spleens of mice were collected, homogenized and passed through 70-μm cell sieve to yield single spleen cell suspension, and red blood cells were dissolved with red blood cell lysate. Spleen cells were re-suspended in 10% inactivated FBS RPMI-1640 medium supplemented with 100 U/mL of penicillin G and 100 U/mL of streptomycin sulfate. Cells were counted and cultured (5 × 10^6^ cells/well) in 24-well plates and stimulated with rPgdA (5 μg/mL) or concanavalin A (5 μg/mL) at 37°C with 5% CO2. At the time of 72 h, the cell supernatant was collected and the levels of cytokines IFN-γ, IL-4, IL-17a, and IL-10 were detected by ELISA kit (BioLegend) according to the instructions.

### Challenge

In the colonization model, bacterial challenge was conducted 2 weeks after the final immunization. Mice were anesthetized with pentobarbital and intranasally challenged with the serotype 19F strain (1 × 10^8^ CFU, 30 µL per nostril). Three days post-challenge, nasal cavity lavage fluid and lungs were collected, serially diluted, and plated on blood agar to count the colonies.

To further evaluate the protective effect of rPgdA against invasive diseases, we utilized both sepsis and pneumonia models. Two weeks after the last immunization, mice in each group were challenged with the D39 strain. In the sepsis model, mice were challenged intraperitoneally with approximately 600 CFU of D39 in 100 µL of sterilized PBS. In the pneumonia model, mice were anesthetized with pentobarbital and challenged intranasally with approximately 1 × 10^8^ CFU of D39 in 30 µL of sterilized PBS. Mouse survival was monitored every 24 h for 21 consecutive days post-challenge.

### Detection of antibodies to rPgdA in human sera

Serum samples from 54 healthy adults and 26 patients with acute pneumococcal pneumonia were collected from the First Affiliated Hospital of Chongqing Medical University. The study excluded people with human immunodeficiency virus, hepatitis B or C virus infection, acute diseases, and severe chronic diseases. The titers of PgdA-specific IgG antibodies in all serum samples were detected by ELISA (as shown in above). This study was approved by the ethics committees of Chongqing Medical University and First Affiliated Hospital of Chongqing Medical University. The informed consent of all participants has been obtained.

### Statistical analysis

All statistical analyses were performed with Graph-Pad Prism 8.0 software. Antibody titers were compared by Mann-Whitney *U* test (two-tailed). Comparisons of the cytokine levels and numbers of CFU were performed with the two-tailed Student’s *t*-test. Survival data were analyzed with the use of the log rank (Mantel–Cox) test. Significant difference was defined as *P* < 0.05.

## Results

### Antibody response in mice immunized with rPgdA

**FIGURE 1:**
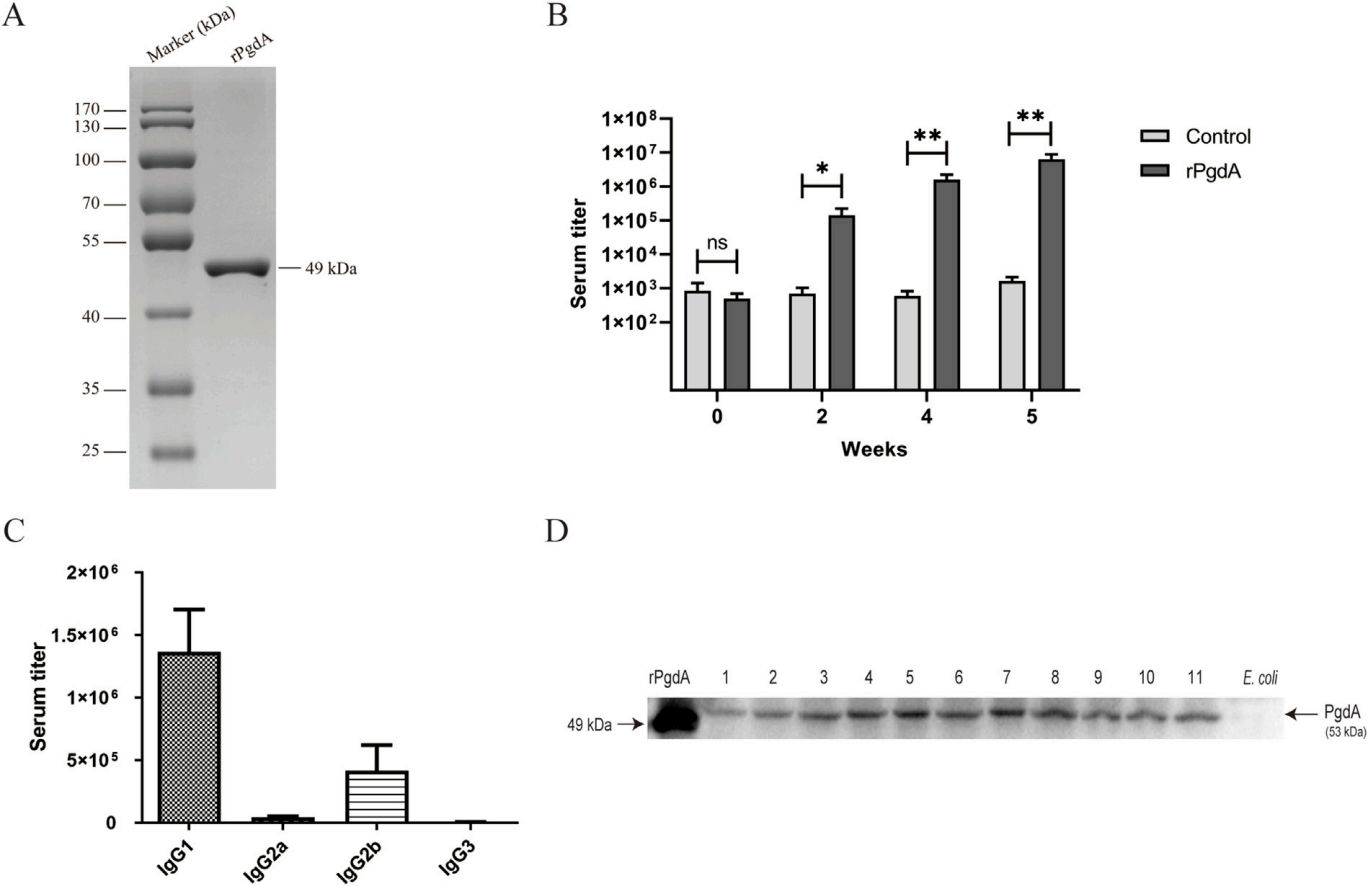
Protein expression and antibody response. **(A)** Analysis of the rPgdA protein by SDS-PAGE. **(B)** The titer of rPgdA-specific total IgG. Sera were collected before each vaccination and 1 week after the last vaccination, and its titer was determined by ELISA. **(C)** The IgG subtypes of rPgdA antiserum were detected by ELISA at 1 week after the final immunization. **(D)** Determination of PgdA in different serotypes of *Streptococcus pneumoniae* by Western blot. Lane 1, serotype 1; lane 2, serotype 2; lane 3, serotype 3; lane 4, serotype 4; lane 5, serotype 6B; lane 6, serotype 7F; lane 7, serotype 9V; lane 8 serotype 14; lane 9, serotype 18C; lane 10, serotype 19F; and lane 11, serotype 23F. The data are presented as means ± SDs, and statistical significance was determined using the Mann-Whitney U test. *p < 0.05; **p < 0.01; ns, not significant.

To evaluate the immunogenicity and the antigen-specific responses of recombinant rPgdA, we first obtained the unlabeled rPgdA protein by affinity purification and determined the molecular weight of rPgdA to be approximately 49 kDa by SDS-PAGE (Figure 1A). Subcutaneous immunization with rPgdA in combination with Alum adjuvant produced a higher IgG antibody titer than adjuvant alone (Figure 1B), demonstrating the activation of humoral immune response. Figure 1C shows that the IgG isotypes produced by rPgdA immunization are mainly IgG1 and IgG2b. These results suggest that rPgdA has good immunogenicity *in vivo*.

To determine whether rPgdA antisera can recognize PgdA antigens, we performed a western blot assay to detect PgdA expression in different serotypes of *S. pneumoniae*. The results showed that rPgdA antisera could specifically recognize the PgdA protein in various *S. pneumoniae* serotypes (Figure 1D). This demonstrates that PgdA is highly conserved across different strains, suggesting that immunization with rPgdA could potentially provide broad protection.

### Effect of rPgdA antisera on bacterial adhesion and invasion to A549 cells

**FIGURE 2:**
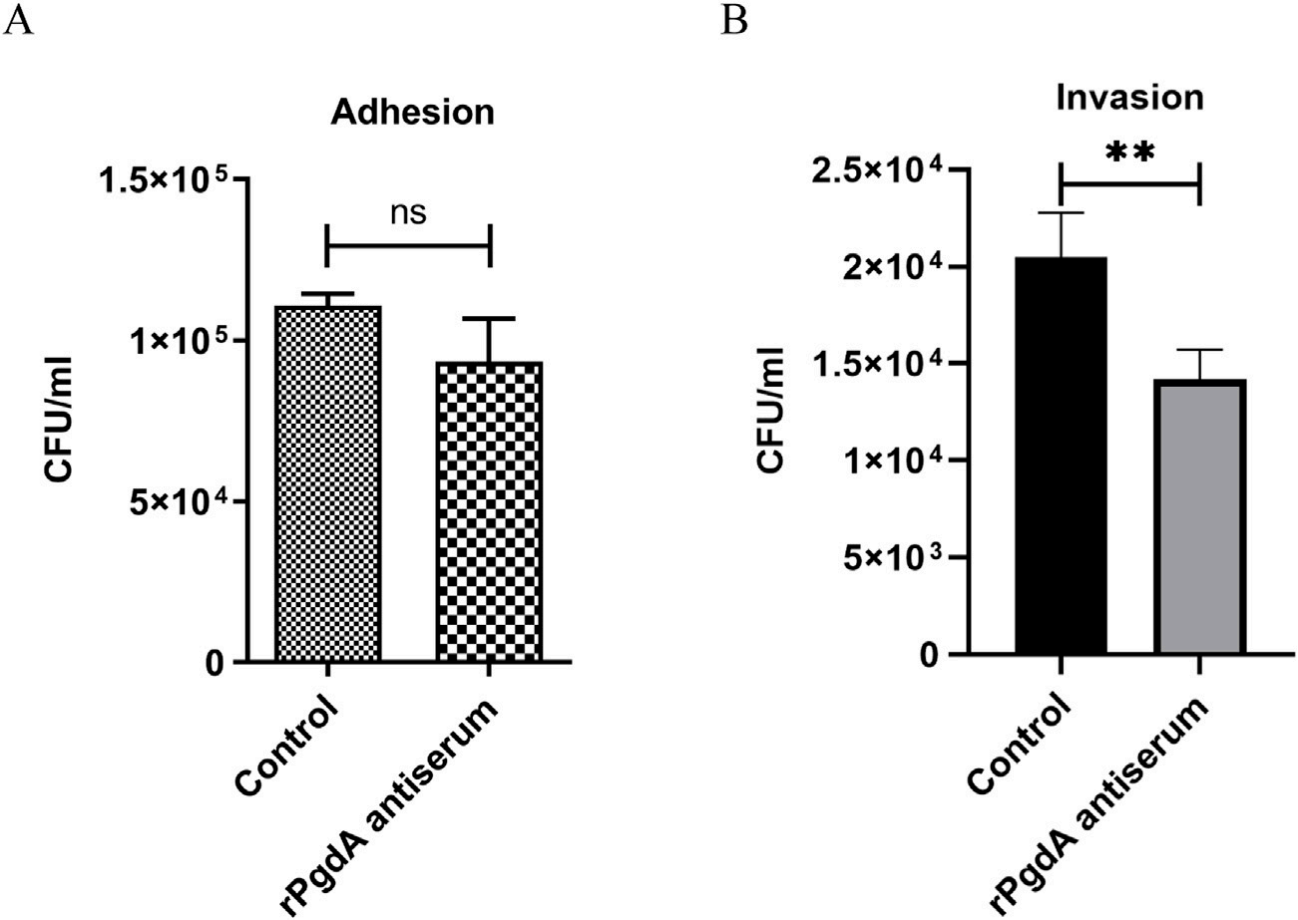
Effect of rPgdA antisera on pneumococcal adhesion **(A)** and invasion **(B)** to A549 lung epithelial cells. TIGR4 bacteria were opsonized with pooled sera from mice vaccinated with adjuvanted rPgdA and then incubated with A549 cells. Sera from mice inoculated with the adjuvant alone served as the control. Error bars represent the results from at least three independent experiments using the same sera. Statistical analysis was analyzed by Student’s *t*-test. **p < 0.01; ns, not significant.

In order to evaluate whether the rPgdA antisera has a certain protective function, we investigated whether the antisera can block the adhesion and invasion of pneumococcus to lung epithelial cells. The results showed that although rPgdA antisera had no significant effect on reducing the adhesion of TIGR4 to A549 cells, it could significantly reduce the TIGR4 bacterial invasion of A549 cells (Figures 2A, B).

### Cytokine production by mouse splenocytes

**FIGURE 3:**
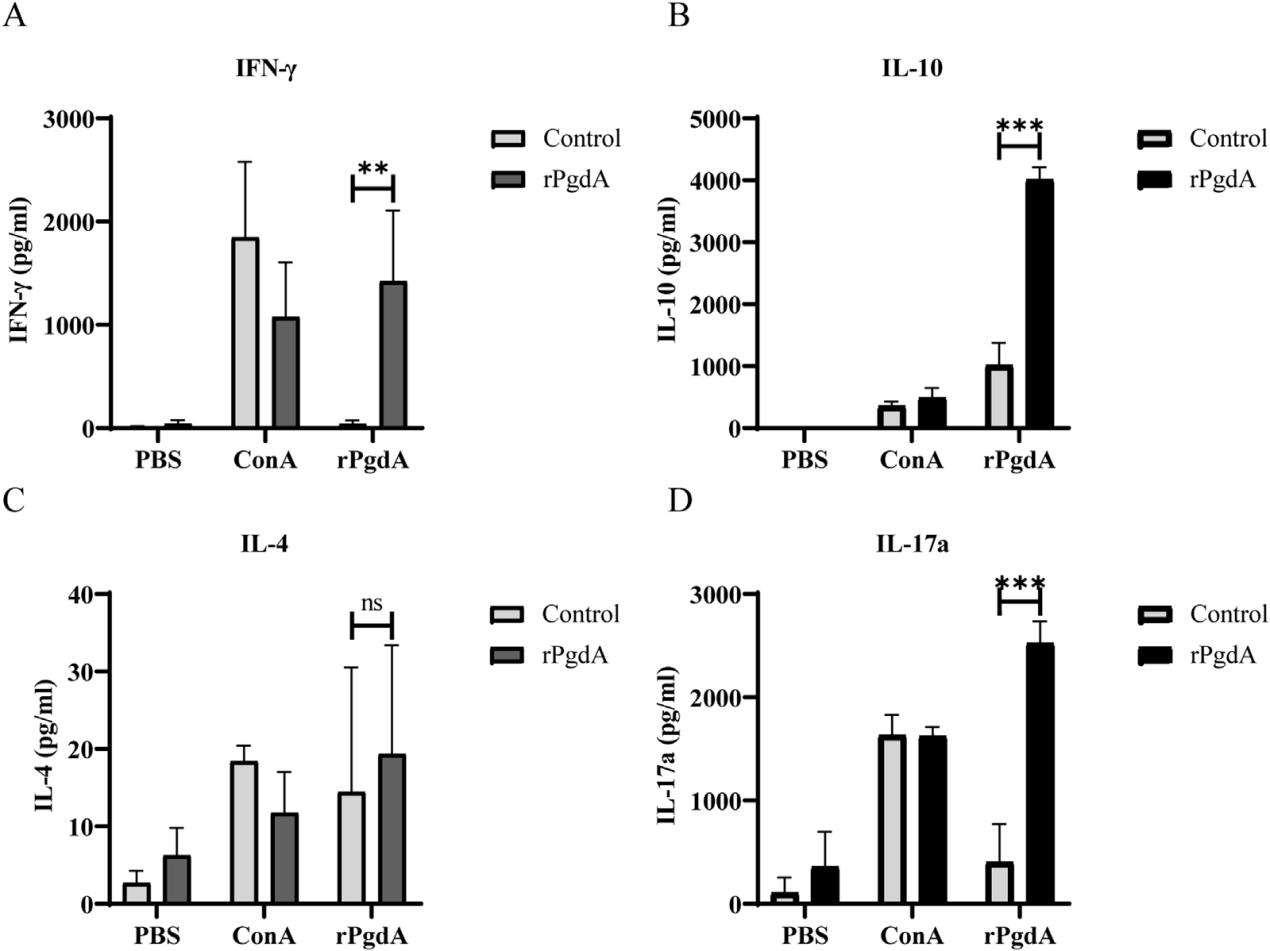
The impact of subcutaneous immunization with rPgdA on cytokine production in murine splenocytes was assessed. Splenocytes were isolated from mice immunized with either adjuvanted rPgdA or adjuvant alone (Control) and analyzed for the levels of IFN-γ **(A)**, IL-10 **(B)**, IL-4 **(C)**, and IL-17a **(D)** using ELISA on day 14 following the final vaccination. The isolated splenocytes were subsequently stimulated with PBS, Concanavalin A (ConA), or rPgdA, with ConA serving as the positive control and PBS as the negative control. The results are presented as means ± SDs. Statistical significance was performed by Student’s *t*-test. **p < 0.01; ***p < 0.001; ns, not significant.

To study the cytokine response induced by rPgdA vaccination, the spleen cells of immunized mice were obtained and stimulated with rPgdA, and the cellular immune responses was evaluated by detecting the production of IFN-γ, IL-4, IL-17a, and IL-10. The results showed that after rPgdA stimulation, significant production of IFN-γ, IL-10, and IL-17a was observed compared with the control group (Figures 3A, B, D). In contrast, no significant change in IL-4 was detected (Figure 3C). These results suggesting that immunization of rPgdA can specifically induce Th1, Th17 as well as regulatory T cell type immune responses.

### Capacity of rPgdA to elicit protection

**FIGURE 4:**
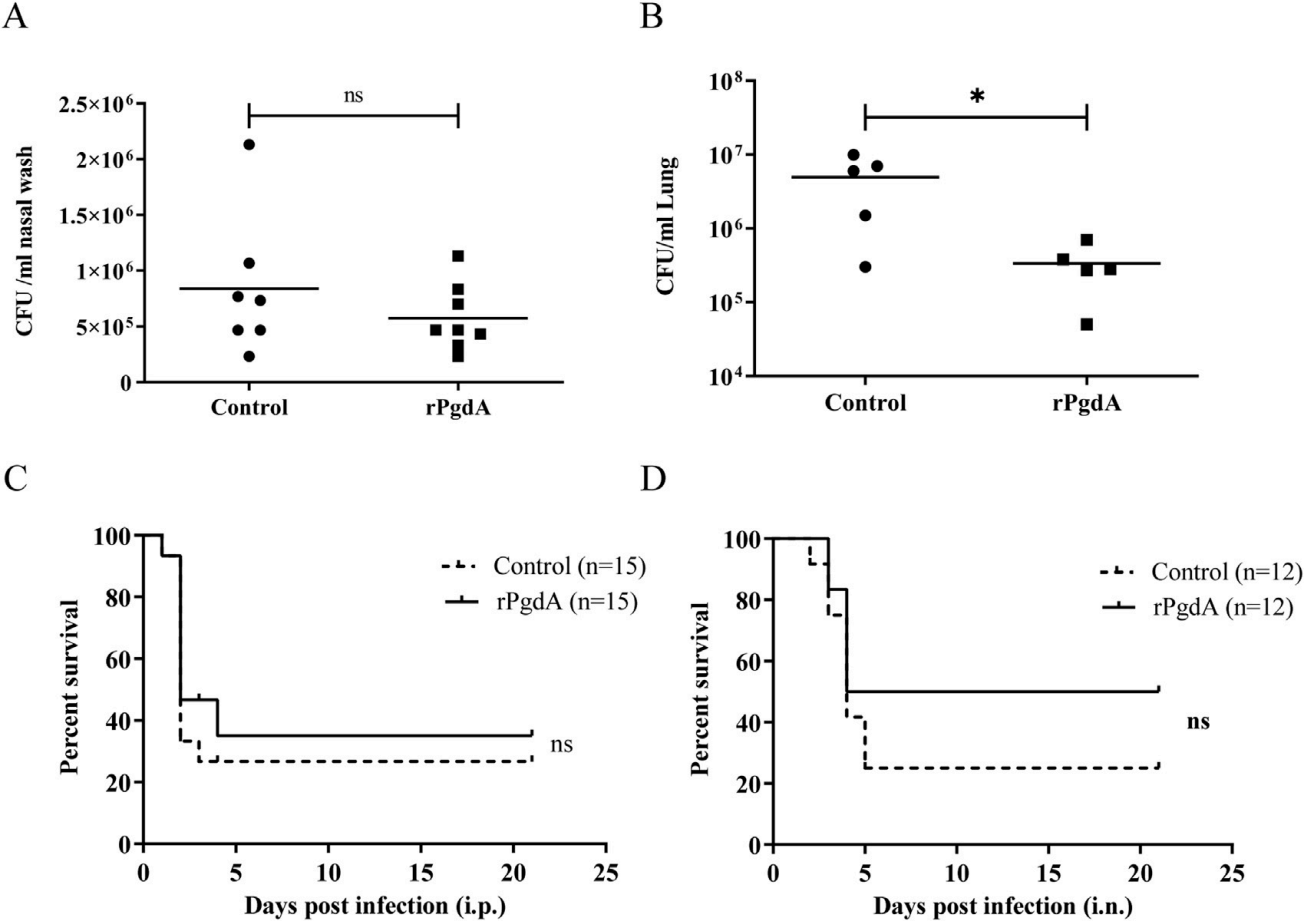
Effect of rPgdA vaccination on pneumococcal challenge. Levels of bacterial load in nasopharynx **(A)** and lungs **(B)** of mice challenged intranasally with *Streptococcus pneumoniae* 19F. The CFUs of nasal colonization and lung invasion were calculated by plating serial dilutions of pneumococci recovered from the nasal washes or lungs, respectively. Each point represents the number of CFU recovered from a mouse. The horizontal line symbolizes the median of each group. Mice immunized with adjuvanted rPgdA or adjuvant alone were challenged with D39 via intraperitoneal **(C)** or intranasal **(D)** route at a dose of 600 CFU and 1 × 10^8^ CFU, respectively, and the survival rates were recorded daily. Statistical significance of survival was analyzed by the log rank (Mantel-Cox) test. *p < 0.05; ns, not significant.

We used the colonization model induced by serotype 19F to evaluate the protective effect of rPgdA on invasive diseases. As shown in Figure 4A, the nasal bacterial burden was comparable between the rPgdA vaccination group and the control group. However, rPgdA-immunized mice had significantly fewer pneumococci in their lungs compared to the adjuvant-treated mice (Figure 4B).

To further evaluate the protective ability of rPgdA, we employed a mouse model of invasive pneumococcal infection. After intraperitoneal challenge with the D39 strain, the survival rate of mice inoculated with rPgdA did not differ substantially from the control group (Figure 4C). In the pneumonia model, mice inoculated with rPgdA and challenged with D39 exhibited improved survival compared to control mice, with survival rates of 50% in the immunized group versus 25% in the control group (Figure 4D). Although this increase in survival rate was not statistically significant, it suggests that rPgdA immunization may reduce the invasion of pneumococci into the lungs without affecting colonization in the nasal cavity.

### Human sera contain antibodies to rPgdA

**FIGURE 5:**
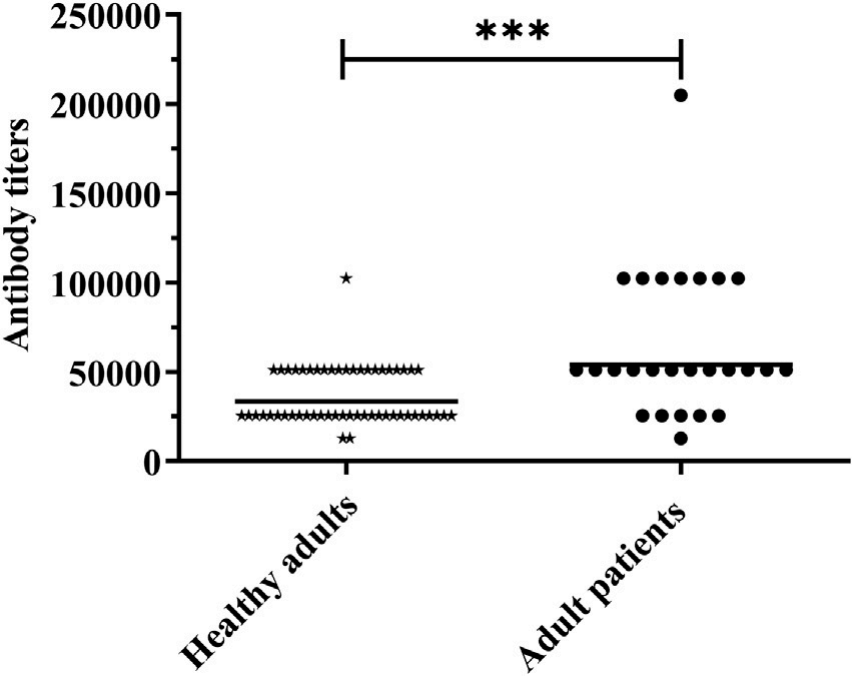
Human serum samples were detected for antibody titer against rPgdA by ELISA. The horizontal line symbolizes the geometric mean of the antibody titer, and each point represents the data of an individual. The significance of the difference was calculated using the Student’s *t*-test. ***p < 0.001.

A previous study showed that the antisera from healthy adults could react with PgdA [26]. To further investigate the immune response to PgdA in humans, we assessed the levels of PgdA-specific IgG in the sera of both healthy adults and patients with acute pneumococcal pneumonia. As illustrated in Figure 5, the geometric mean titer of serum IgG antibodies against PgdA was significantly higher in patients with acute pneumococcal pneumonia compared to healthy adults. This finding suggests that a natural immune response to PgdA is elicited during pneumococcal infection.

## Discussion

In this study, our results demonstrated that rPgdA has good immunogenicity, and immunization with rPgdA can induce robust systemic immune response in mice. In addition, we found that subcutaneous immunization of mice with rPgdA did not significantly affect the colonization of pneumococcus in the nasopharynx, but could significantly reduce the invasion of pneumococcus in the lung. More importantly, we found that PgdA antibodies may play a role in the prevention of pneumococcal disease in humans.

Previous studies have shown that incorporation of affinity tag to a recombinant protein may alter its structure, change its immunogenicity, and cause non-specific reactions [27–30]. To reduce these negative effects, we used the Profinity eXact protein purification technology in this study, which helps to purify recombinant proteins with natural N-terminus. Through subcutaneous immunization of mice with unlabeled rPgdA, we can obtain antibodies that recognize the PgdA protein of multiple pneumococcal serotypes, which indicates that the recombinant PgdA protein may possess broadly conserved epitopes, potentially providing extensive protection.

In the colonization model, we found no significant difference in the colonization level of 19F after rPgdA inoculation compared with the adjuvant group alone, but a significant reduction in the bacterial burden in the lungs. We reason that the lack of protective effect of rPgdA immunization on colonization may be due to the blocking effect of pneumococcal capsular polysaccharide on antibodies, which has been demonstrated by Zangari, T., et al. [31]. However, it has a protective effect on lung colonization, which may be due to the following reasons. Firstly, a previous study showed that in order to effectively invade cells, *S. pneumoniae* will reduce the content of capsular polysaccharide to fully expose adhesion molecules [32]. At the same time, one of our previous studies showed that PgdA would be induced to express in the lungs [25], thus the expression of PgdA is increased during invasion. The above factors can increase the chance of PgdA antigen exposure and recognition by its antisera. Secondly, our results suggest that rPgdA antisera can reduce pneumococcal invasion of lung epithelial cells. Finally, we cannot ignore the role of cellular immune responses, as our cytokine analysis showed that the levels of IL-17A, IL-10, and IFN-γ were significantly increased in splenocytes stimulated with rPgdA. As other researchers have shown, these cellular immune responses are critical for defense against pneumococcal infections, and a comprehensive immune response is necessary for effective elimination of *S. pneumoniae* [33–36].

Th17 responses are crucial for controlling nasal colonization and preventing invasive disease in pneumococcal infections. Th17 cells play a significant role in clearing *S. pneumoniae* from the nasopharynx by enhancing neutrophil recruitment and promoting local inflammation [37]. Despite the robust Th17 responses observed in mice vaccinated with rPgdA, these animals did not show a reduction in nasal colonization. Several factors may contribute to this discrepancy. First, although rPgdA vaccination induces a Th17 response, this response alone may be insufficient to counteract the specific mechanisms facilitating pneumococcal colonization. The vaccine might stimulate effective immunity against invasive disease but be less adept at targeting nasal colonization due to the complex interactions between pneumococcal surface proteins and the host immune system [38, 39]. Additionally, *S. pneumoniae* has evolved various immune evasion mechanisms, such as modulating surface antigens and evading phagocytosis, which allow it to persist in the nasopharynx [40–42]. Even with a robust Th17 response, these evasion strategies could enable pneumococci to maintain colonization. Therefore, the rPgdA vaccine might not fully address these evasion mechanisms, leading to persistent nasal colonization despite the Th17 response.

In the survival challenge model, rPgdA inoculation did not exhibit a protective effect against an intraperitoneal challenge, but there was an improvement in survival in the pneumonia model, although this increase was not statistically significant. Adequate protection was not observed in this study, potentially due to the pneumococcal capsule’s ability to block antibody function, as suggested by a recent study [31]. Another factor could be the differing immune environments in the abdominal cavity and lungs. The abdominal cavity, primarily a sterile environment, lacks the mucosal immune components found in the lungs, which are constantly exposed to various inhaled pathogens and particles. This disparity in immune environments might affect the efficacy of the vaccine, with the lungs being better equipped to mount a robust local immune response.

We analyzed serum samples from adults with invasive pneumococcal infections and found that antibody titers against PgdA were significantly higher compared to those in healthy adults. This suggests that PgdA may be involved in the pneumococcal infection process. The elevated levels of anti-PgdA antibodies in patients with invasive pneumococcal infections imply that the immune response to PgdA could play a role in combating or preventing such infections.

In conclusion, subcutaneous immunization with rPgdA can induce humoral and cellular immunity, which can reduce the invasion of *S. pneumoniae* to the lung. Simultaneously, no discernible effect on colonization was observed in our experimental setting. Considering the limited improvement in survival, we recommend its use in combination with other proteins for better prevention of pneumococcal infection under the new strategy.

## Data Availability

The original contributions presented in the study are included in the article/supplementary material, further inquiries can be directed to the corresponding author.

## Footnotes

1
http://www.cbs.dtu.dk/services/TMHMM/
